# Exercising Increased Caution Before Removing C-collars in Altered Patients and Patients With Chronic Cervical Spine Changes: Reports of Neurological Deficit or Injury Following C-collar Removal Despite Normal Imaging

**DOI:** 10.7759/cureus.84054

**Published:** 2025-05-13

**Authors:** Payton C O'Quinn, Lou M Smith

**Affiliations:** 1 Department of Surgery, East Tennessee State University Quillen College of Medicine, Johnson City, USA; 2 Department of Surgery, University of Tennessee Medical Center, Knoxville, USA

**Keywords:** acute care surgery and trauma, cervical spinal cord injury without radiographic abnormalities, cervical spinal injury, cervical spine clearance, surgery, trauma

## Abstract

Removing a cervical collar (C-collar) in trauma patients is a clinically complex and often controversial subject; existing guidelines have some areas of ambiguity. Little to no literature exists regarding C-collar removal in patients with chronic degenerative changes of the cervical spine or dementia. We present two trauma patients who were initially cleared of their C-collars after undergoing a cervical spine CT scan. Both patients later required their C-collars to be replaced and required neurosurgical consultation; one patient developed neurological deficits. Both patients were mentally altered in some form; one via intoxication and the other via existing dementia. Additionally, both patients had chronic degenerative cervical spine changes. While adverse outcomes following C-collar removal are rarely recorded in the literature, this is likely due to underreporting. In the absence of literature specific to chronic degenerative changes or dementia, we recommend that clinicians exercise increased caution when removing C-collars in this patient population.

## Introduction

The decision to remove a cervical collar (C-collar) in a trauma patient is a complex and often contentious clinical decision that is guided by existing literature. In general, the current guidelines recommend that C-collars should be removed as soon as is medically feasible [1]. The Eastern Association for the Surgery of Trauma (EAST) recommends clinical clearance when possible; alert, cooperative patients without neck pain, distracting injury, or neurological deficits can have their C-collar removed without imaging [1]. All other patients should undergo a cervical spine CT scan to assess for acute injuries or abnormalities; the guidelines indicate that patients with a normal CT scan can have their C-collars removed with almost no risk of adverse outcomes [2,3]. The Western Trauma Association echoes EAST, encouraging clinical clearance of the cervical spine when possible and use of CT imaging in patients with a potentially unreliable physical exam [4]. A recent study found that a change in Glasgow Coma Scale was the most common cause of an unreliable exam, demonstrating the increased difficulty of removing C-collars in altered patients [5]. There is also literature to suggest CT scans are effective at detecting ligamentous injury. Duane et al. performed a prospective study of over 500 patients with traumatic cervical spine fractures and found zero patients with a “normal” CT who were later found to have ligamentous injury [6]. However, what constitutes a “normal spine CT” has not been vigorously defined. The guidelines also recommend exercising increased caution when removing C-collars in patients with altered mental status or distracting injuries, but these terms are also variably defined. There is considerable literature regarding the removal of C-collars in obtunded patients, but little to no literature regarding dementia or other types of altered mental status patients with C-collars [7]. Inconsistency in definitions and practice has led to discordant recommendations in the surgical literature. Here, we present two trauma patients who were initially cleared of their C-collars after undergoing a cervical spine CT scan. Both patients later required their C-collars to be replaced and required neurosurgical consultation; one patient developed neurological deficits. IRB approval was granted for the publication of both patient cases.

## Case presentation

**Figure 1:**
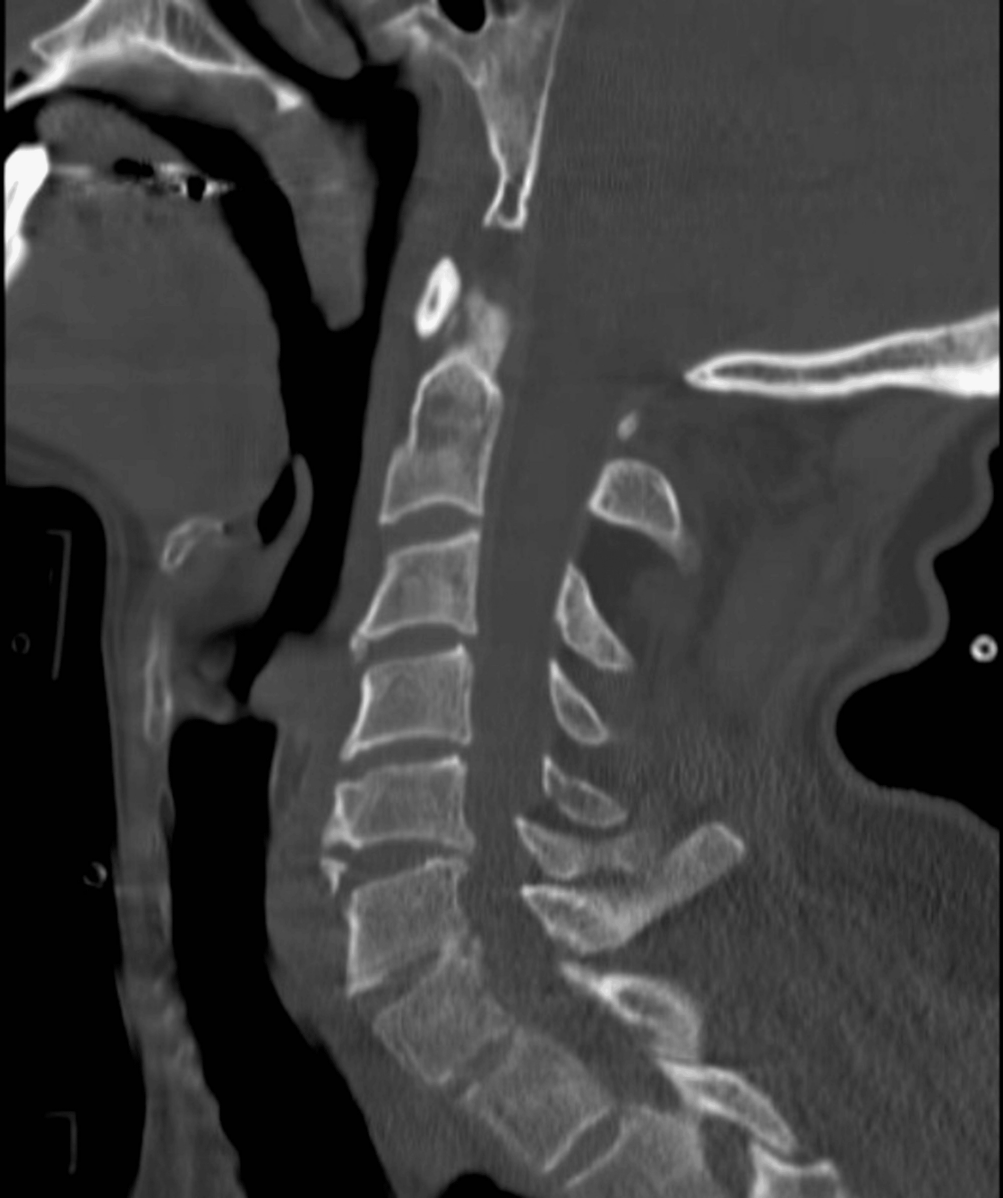
Sagittal view of the CT cervical spine, read as normal with chronic degenerative changes

The first patient was a 51-year-old man with no significant past medical history who presented to the trauma bay as a blunt trauma patient following a motorcycle crash in which the patient hit a pole. The patient was in a C-collar upon arrival in the trauma bay. He was noted to be intoxicated but hemodynamically stable. The primary trauma exam was normal; the secondary exam was notable for a lethargic affect, pinpoint pupils, and a normal sensorimotor. GCS was 13 with a verbal score of 3. A right femoral neck fracture was also noted. The patient underwent a CT of the cervical spine, which was without acute findings but did reveal chronic degenerative changes of the cervical spine (Figure 1).

**Figure 2:**
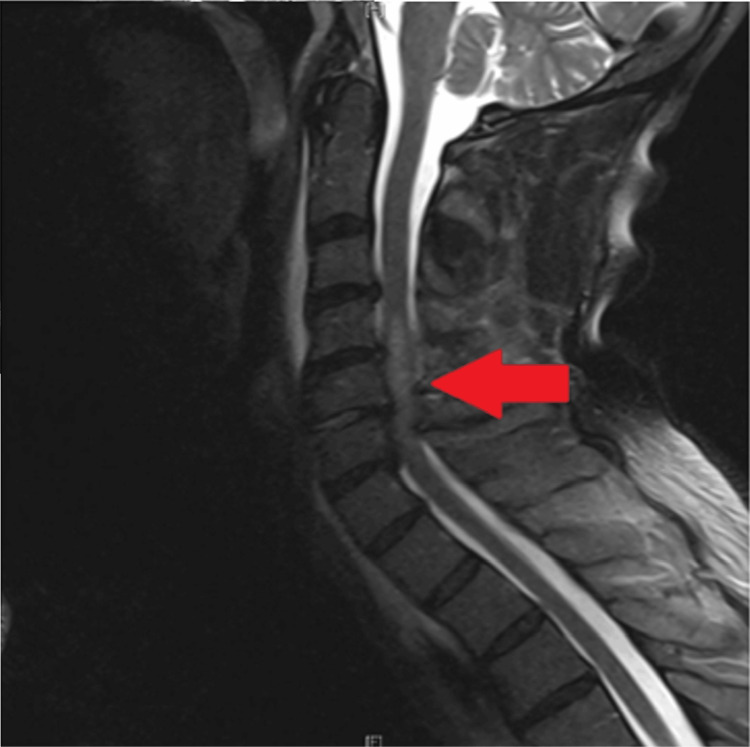
Multiplanar multisequence MRI cervical spine performed without intravenous contrast revealing cervical cord edema, as shown by the arrow; anterior longitudinal ligament disruption can be seen at C4-C5

Following this, the patient was clinically cleared of his C-collar and transferred to the surgical intensive care unit. Overnight and within six hours of his C-collar being removed, the patient began complaining of sensorimotor loss in his bilateral lower extremities, along with motor loss in his bilateral upper extremities. The neurosurgery team was consulted at this time, and the C-collar was replaced. The patient underwent MR imaging, which revealed cervical cord edema at the level of C4-C7 (Figure 2).

The MRI additionally revealed disruption of the anterior longitudinal ligament (ALL) at C5-C6. The patient was taken to the operating room by the neurosurgery team, who performed a posterior cervical decompression and fusion. The patient remained intubated and in a C-collar in the immediate postoperative period. He was extubated successfully on postoperative day 2 and was transferred to the progressive care unit on postoperative day 3, before being transferred to a medical-surgical floor on postoperative day 25. The patient required prolonged hospitalization and the use of a C-collar. The patient began refusing to wear his C-collar on postoperative day 67. His neurological status remained largely unchanged, continuing with incomplete quadriplegia. He subsequently transferred to a rehab facility. We do not have long-term follow-up. 

**Figure 3:**
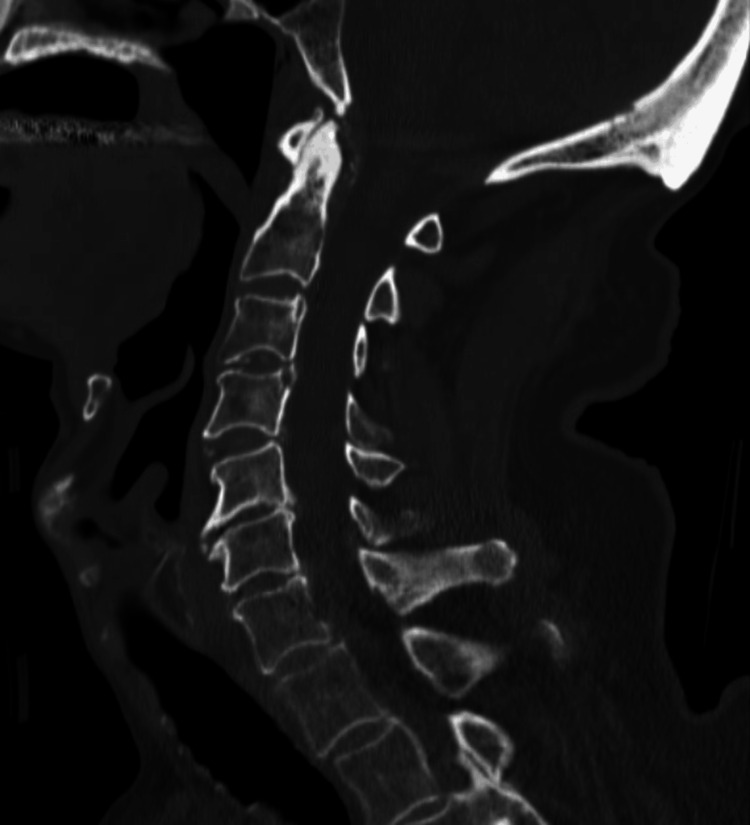
Sagittal view of the CT cervical spine, read as normal with chronic degenerative changes

The second patient was an 85-year-old man with a medical history of atrial fibrillation on amiodarone and with a pacemaker. They presented to the trauma bay within the 90-day window of the first patient. The patient also had dementia at baseline. The patient experienced a ground-level fall at his senior living facility and was transferred to the hospital as a blunt trauma patient without cervical immobilization. He had a GCS of 15 on arrival. The primary trauma survey was notable for being on 2 liters of oxygen via nasal cannula. Secondary survey was notable for lacerations and ecchymosis to the face, normal sensorimotor exam in the upper and lower extremities, and baseline confusion. A C-collar was placed in the trauma bay. CT of the cervical spine showed chronic degenerative changes of the cervical spine with no acute findings (Figure 3), but CT of the head showed a subarachnoid hemorrhage.

**Figure 4:**
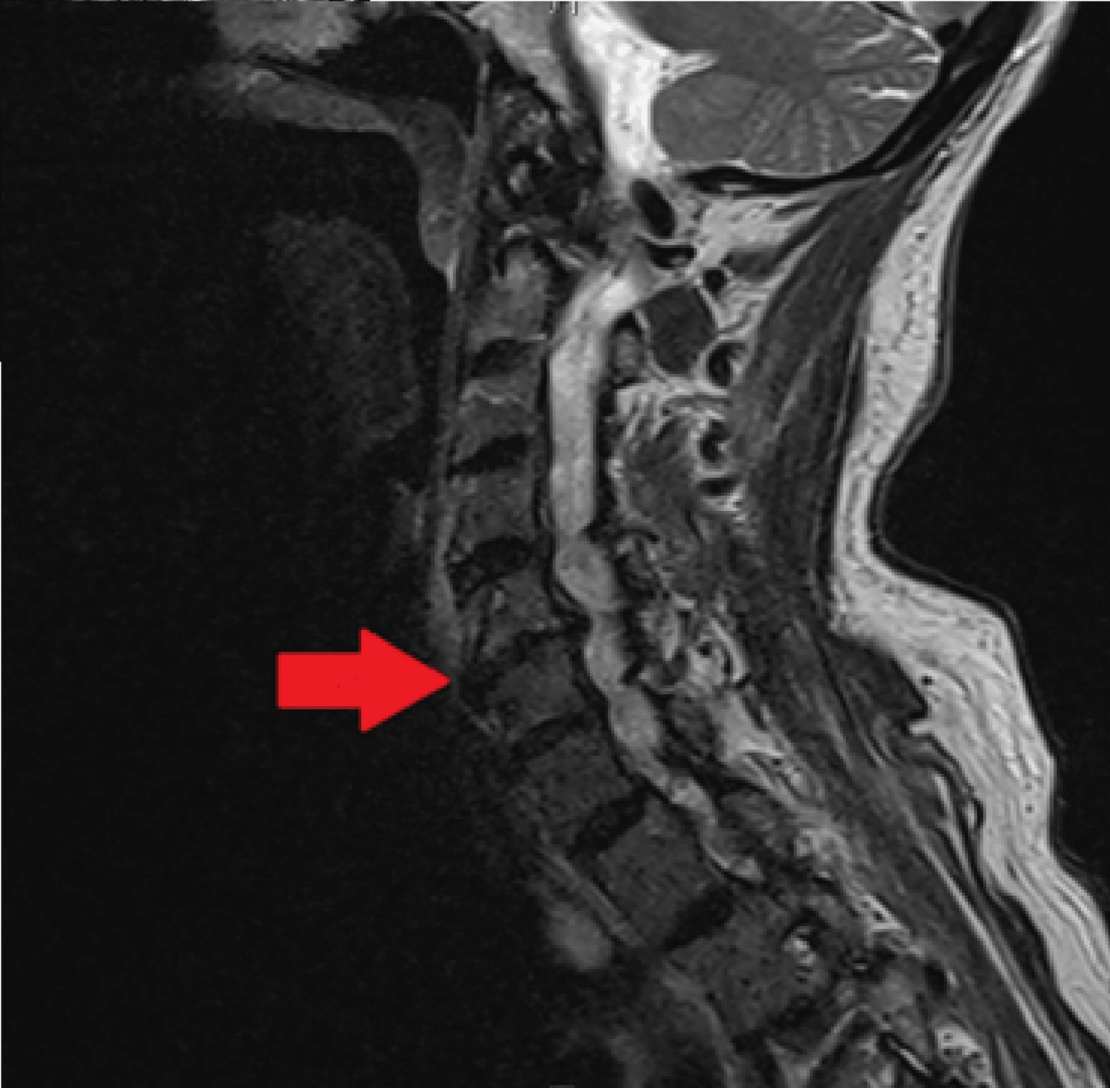
Multiplanar multisequence MRI cervical spine performed without intravenous contrast revealing partial thickness tear of the anterior longitudinal ligament tear, as shown by the arrow

The C-collar was removed in the emergency department after a negative clinical examination but was reapplied shortly after at the patient’s daughter’s request. The patient continued to experience altered mental status with a GCS of 14. The following morning, the neurosurgery team saw the patient for further evaluation of the subarachnoid hemorrhage. During their consultation, the neurosurgeon raised concerns regarding possible ligamentous injury at C4-C5 based on his interpretation of the initial CT of the cervical spine. Notably, the neurosurgeon’s read of the imaging differed from the official radiologist's interpretation. Neurosurgery recommended maintaining the C-collar at all times; an MRI of the brain and cervical spine revealed a partial thickness tear of the ALL at C4-C5 (Figure 4).

Notably, the patient did not experience any neurological deficits from his ligamentous injury and had only a short period out of his collar. Unfortunately, the patient was progressively more altered from baseline due to his traumatic brain injury. The decision was made by the patient’s family to transfer him to comfort care. The patient was transferred to inpatient hospice on hospital day 13 and expired on hospital day 18.

## Discussion

Current guidelines recommend clearing C-collars as soon as medically possible but encourage increased caution in patients with distracting injuries or altered mental status. However, numerous aspects of the recommendations are not clearly defined. There is no consistent guideline regarding C-collar removal in patients with baseline dementia, alcohol intoxication, or findings of preexisting degenerative disease of the cervical spine without acute fractures. The majority of research regarding C-collar removal in patients with altered mental status relates to obtunded patients with traumatic brain injuries. Treating trauma patients with dementia or baseline neurological changes is especially challenging for clinicians, as it may hamper the reliability of a clinical exam. We conducted a PubMed search using terms such as “dementia,” “cervical collar,” “dementia & cervical collars,” “cervical spine clearance,” “chronic cervical changes,” and “chronic degenerative disease & cervical collars” and were unable to find any literature or guidelines specific to either of these topics. There is research to support the removal of C-collars in intoxicated patients. The Western Trauma Association published a multi-institutional trial and survey indicating that CTs of the cervical spine have a high degree of accuracy in detecting acute abnormalities, even in intoxicated patients, and recommended early C-collar discontinuation for normal CTs [8].

The patient in our first case was intoxicated and had a potentially distracting injury, while the patient in the second case had baseline dementia. Both patients had chronic degenerative cervical spine findings. The second patient did not develop any neurological deficit from his ALL tear but was only out of his C-collar for a short period of time, even after the negative CT of the cervical spine. It is notable that current guidelines would have recommended this patient's C-collar be completely removed, while he ultimately required the collar for the remainder of his hospitalization. Both patients had negative clinical outcomes despite adherence to the existing guidelines. Despite guidelines recommending C-collar clearance in intoxicated patients with a normal CT, it is clear that intoxication hampers the clinical exam. Patient one's collar was removed in accordance with the EAST guidelines but required his C-collar to be replaced within 24 hours of removal. It is possible that the injury had not fully developed at the time of CT, but regardless, we believe that this raises some concern about gaps within the existing literature.

These cases fall in a potential gray area of the existing guidelines and demonstrate how many clinically complex situations frequently do not fall neatly within the guidelines and may result in adverse outcomes. Furthermore, it is notable that both patients had negative CTs of their cervical spines read by a board-certified radiologist, with later imaging revealing acute injuries. While spinal cord injuries without radiographic abnormalities (SCIWORA) are rare, they are not unheard of. Oto et al. performed a review of the literature suggesting that while neurological deterioration following blunt spinal trauma is infrequent, particularly after C-collar removal, it is probable that such occurrences are underreported for liability and other reasons [9]. This may instill a false sense of safety in clinicians tasked with the evaluation of blunt trauma patients. Furthermore, neurological deterioration following blunt spinal injury is rarely reported in the literature, leading to a possible lack of clarity in guidelines and recommendations when it does occur [9]. While the majority of guidelines continue to recommend removing the C-collar after a normal CT of the cervical spine, it should be noted that CT scans are less sensitive than MRIs when detecting ligamentous injuries [10]. However, prior studies have not supported routine use of MRI to detect ligamentous injuries, as these findings do not always alter clinical management [7]. Additionally, no imaging study exists that detects 100% of SCIWORA.

## Conclusions

Removal of C-collars in trauma patients remains a controversial and contentious subject, with conflicting recommendations. There is currently a lack of research regarding dementia and chronic cervical spine changes in patients with C-collars. We have presented two patients who underwent guideline-directed clearance of rigid C-collars; both patients experienced clinical decline and required C-collar replacement. The first patient progressed to incomplete quadriplegia, and the second patient expired from a concurrent traumatic brain injury. It is notable that both patients had some form of altered mental status, with the first patient arriving intoxicated and the second patient having baseline dementia. We recommend that clinicians exercise increased caution when removing C-collars in patients who are intoxicated or have any neurological condition, such as dementia or delirium, that affects reliable examinations. Degenerative disease of the cervical spine may also be a marker for increased risk of SCIWORA. 

We believe that further study is necessary to elucidate how dementia and chronic changes of the cervical spine should impact C-collar clearance. We hope that sharing our clinical experiences will encourage clinicians to continue to use cautious clinical judgement, report adverse outcomes to increase awareness and potential research, and be wary of any alteration in mental condition when making the decision to remove a C-collar. 
